# Life stressors, hypertensive disorders of pregnancy, and gestational diabetes by race/ethnicity

**DOI:** 10.1371/journal.pone.0321615

**Published:** 2025-04-21

**Authors:** Forgive Avorgbedor, Thomas P. McCoy, Amita Mittal, Lori Hubbard, Stephanie Pickett

**Affiliations:** School of Nursing, University of North Carolina Greensboro, North Carolina, United States of America; Universita degli Studi dell'Insubria, Italy

## Abstract

**Background:**

In the United States, adverse pregnancy outcomes, including hypertension before pregnancy (HTN), pregnancy-induced hypertension (PIH) [gestational hypertension and preeclampsia] and gestational diabetes mellitus (GDM) continue to increase. Stressful life events (SLEs) such as serious illness, divorce, are known to impact adverse birth outcomes, e.g., preterm birth, especially among Black women, low-income women, and other minority women than White women. However, there is limited evidence on SLEs adverse pregnancy outcomes. Therefore, the objective of this study is to provide an overview of trends in stressful life events from 2009 to 2020 and their impacts on hypertension before pregnancy, pregnancy-induced hypertension, and gestational diabetes mellitus in the United States and to understand these effects by race/ethnicity.

**Methods:**

A secondary analysis of Centers for Disease Control and Prevention national Pregnancy Risk Assessment Monitoring System data from 2009 to 2020 was performed. SLEs, HTN before pregnancy, PIH, and GDM, were examined with data visualizations and multivariable weighted log-binomial modeling.

**Results:**

Any SLE prevalence was 66% to 72%, with Black women having higher SLEs than White women. SLE was associated with HTN before pregnancy (ARR = 1.082), PIH (ARR = 1.059), and GDM (ARR = 1.030). Effects of race/ethnicity differed across these outcomes.

**Conclusion:**

Greater SLE is associated with adverse pregnancy outcomes. Black women continue to experience higher SLEs and are at higher risk of HTN before pregnancy and PIH. The findings of this study indicate there is an interplay between SLEs, HTN before pregnancy, PIH, and GDM, as well as race/ethnicity. This information is vital for public health efforts to reduce the disparities in adverse pregnancy outcomes.

## Introduction

In the United States (US), 7 in 10 women reported at least one stressful life event (SLE) a year before delivering a child [1–3]. Adverse maternal outcomes, including hypertension before pregnancy (HTN), and pregnancy-induced hypertension (PIH) [gestational hypertension and preeclampsia] and gestational diabetes mellitus (GDM), continue to increase [4,5]. The prevalence of adverse maternal outcomes is higher among Black women, low-income, and other minority women than White women [6–8]. SLEs, such as divorce, homelessness, or losing a family member, are linked to adverse birth outcomes, including preterm birth and low birth weight [1,9]. However, there are limited studies on the link between SLEs and HTN before pregnancy, PIH, and GDM, using large national data.

GDM and PIH are frequent complications in pregnancy, [10] with growing evidence indicating a potential association between these complications and SLEs [4,10–12]. These complications continue to increase, in part due to increased maternal age, and are associated with short and long-term adverse maternal and infant morbidity [5,13]. However, there is limited literature on SLEs and adverse pregnancy outcomes. Given the emerging evidence on the relationship between SLE and adverse pregnancy outcomes, there is a need to update the literature on the current trends with rigorous data.

Chronic stress is a risk factor for cardiovascular disease and Type 2 diabetes mellitus [14,15]. Hypertensive disorders of pregnancy, including gestational hypertension and preeclampsia, are associated with maternal morbidity and mortality, as well as short- and long-term cardiovascular disease [16]. Additionally, GDM is also associated with the development of Type 2 diabetes mellitus after pregnancy [17,18]. The interplay between adverse pregnancy outcomes and maternal stress is complex. However, establishing a relationship between adverse pregnancy outcomes and maternal stress with large data may lead to future interventions. Therefore, the objective of this study is to provide an overview of trends in stressful life events from 2009 to 2020 and their impacts on hypertension before pregnancy, pregnancy-induced hypertension, and gestational diabetes mellitus in the United States and to understand these effects by race/ethnicity.

## Materials and methods

This study was a secondary analysis of available Pregnancy Risk Assessment Monitoring System (PRAMS) data. PRAMS is a US surveillance program conducted annually by state health departments and the Centers for Disease Control and Prevention (CDC). Random samples of mothers who had a live birth are invited to participate in PRAMS data collection, including a follow-up phone call. If the response rate among those invited mothers is high enough for a given year, then the CDC will include those state data in their US-wide data collection. From 2009 through 2020, PRAMS was conducted in 38–50 sites, but the release of data by site varies by year (range 27–43). Data collection includes self-reported behaviors at prenatal, perinatal, and postnatal stages. More details about PRAMS can be found in Shulman and colleagues (2018) [19]. The study is considered a secondary data analysis for which consent is not required. We have no access to information that could identify individual participants during or after data collection. The general PRAMS methodology and protocol approved by the CDC PRAMS Working Group, and PRAMS participating states health departments local institutional review board of record.

### Measures

#### Stressful life events (SLE).

PRAMS asks questions about SLES 12 months before the infant’s birth. The PRAMS core questionnaire includes 13 (Phase 6 of PRAMS) or 14 (Phases 7–8) questions about the following life events: questions from the phase 7 core questionnaire include: 1) A close family member was very sick and had to go into the hospital; 2) I got separated or divorced from my husband or partner; 3) I moved to a new address; 4) I was homeless or had to sleep outside, in a car, or in a shelter; 5) My husband or partner lost his job; 6) I lost my job even though I wanted to continue working; 7) My husband, partner, or I had a cut in work hours or pay; 8) I was apart from my husband or partner due to military deployment or extended work-related travel; 9) I argued with my husband or partner more than usual; 10) My husband or partner said he didn’t want me to be pregnant; 11) I had problems paying the rent, mortgage, or other bills; 12) My husband, partner, or I went to jail; 13) Someone very close to me had a problem with drinking or drugs; 14) Someone very close to me died. It is common to sum up a count of the Yes responses to the SLEs and also examine the prevalence of at least one SLE in the past 12 months [1]. A one-factor categorical CFA model estimated using weighted least squares estimation revealed good construct validity (RMSEA = 0.064), with factor loadings of 0.4 or higher except for the item: (0.205). Internal consistency was KR-20 = 0.66.

#### Hypertension before pregnancy.

Hypertension (HTN) before pregnancy was measured with a single Yes/No self-reported question: “During the *3 months before* you got pregnant with your *new* baby, did you have any of the following health conditions? High blood pressure or hypertension.”

#### Pregnancy-induced hypertension (PIH).

PIH was measured with a single Yes/No self-reported question: “During your most recent pregnancy, did a healthcare provider tell you that you had any of the following health conditions? “High blood pressure (that started during this pregnancy), preeclampsia, or eclampsia.” PIH was collected in Phases 6 (years 2009–2011) and 8 (2016–2020), but for Phase 7 (2012–2015), only seven (Alabama, Connecticut, Delaware, Hawaii, Illinois, Maine, and Wisconsin) of the 36 participating states (19.4% of states) collected data on PIH. Therefore, we only analyzed data from these states for all three phases of subgroup analysis.

#### Gestational diabetes mellitus (GDM).

GDM was measured with a single Yes/No self-reported question: “During your most recent pregnancy, did a healthcare provider tell you that you had any of the following health conditions? “Gestational diabetes (diabetes that started during this pregnancy)”

#### Race/Ethnicity.

Due to some racial/ethnic groups having smaller unweighted counts, race/ethnicity was categorized into the following four groups for analysis: Black (non-Hispanic), Hispanic, White (non-Hispanic), and All Other (subsequently denoted Other).

#### Covariates.

The following maternal characteristic variables were included: maternal age (years-continuous variable), education level, marital status, BMI, smoking during 3^rd^ trimester of pregnancy, alcohol use prepregnancy, and insurance status.

#### Statistical methods.

Weighted percentages for states and overall, by year, were estimated to investigate trends over time. Multivariable modeling of dichotomous adverse pregnancy outcomes (HTN before pregnancy, PIH, and GDM) was performed using log-binomial modeling [20] to estimate adjusted relative risk ratios (ARRs) and their 95% confidence intervals (CIs). Of *a priori* interest were the interactions between race/ethnicity groups, HTN before pregnancy, and year, when applicable. Subpopulation domain analysis [21] was performed for study inclusion criteria of those with a single parity and maternal age between 18 and 45 (inclusive). Because of subgroup size, multivariable modeling for HTN before pregnancy and PIH was limited to adjusting for SLE, race/ethnicity, and year. All analyses were weighted and adjusted for the PRAMS complex sample design [19]. Collinearity was evaluated for all models using eigenanalysis and variance inflation factors (VIFs). All analyses were performed in SAS v9.4 (SAS Institute, Cary, NC) and STATA v17.0 (STATA Corp., College Station, TX). A two-sided *p*-value < 0.05 was considered statistically significant.

## Results

The study data comprised a total of 452,031 women between the ages of 18 and 45 from 2009 to 2020 (weighted *N* = 22,817,249), including 49% White, 17% Black, 17% Hispanic, and 16% all other race/ethnicity. For the subgroup analysis of those with PIH collected, a total of 65,774 women from seven states from 2009 to 2020 were analyzed (weighted *N* = 2,975,688 women).

**Fig 1:**
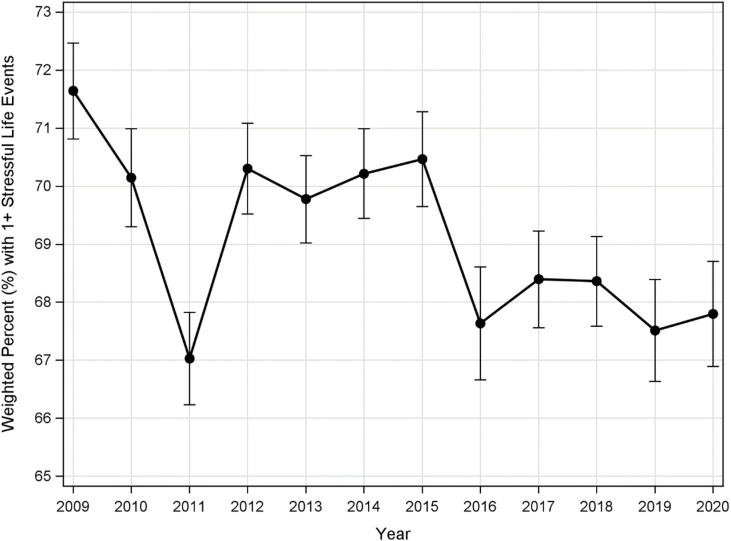
Trend over time in stressful life events (Error Bars = 95% confidence interval).

**Fig 2:**
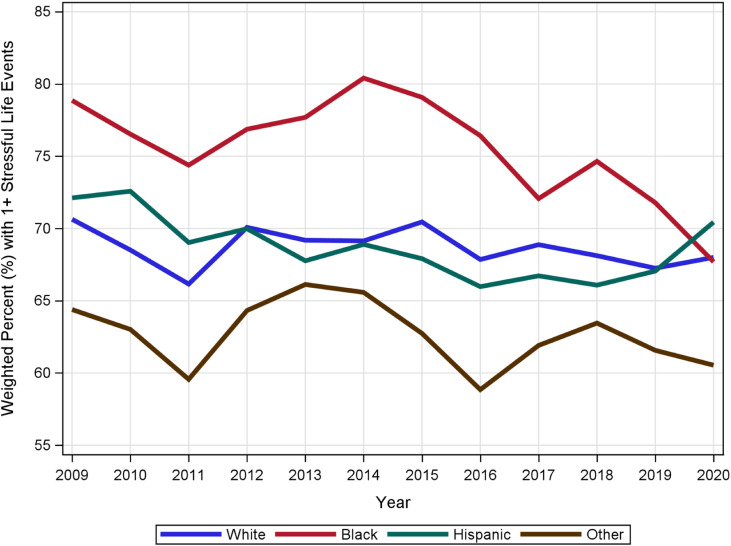
Trends over time in stressful life events by race/ethnicity.

The percent with any SLEs is between 66% and 72% across the years. Fig 1 shows the overall trend in SLEs from 2009 to 2020. Additionally, Fig 2 shows SLEs trends by race/ethnicity group. Black women, in particular, had higher weighted SLE rates for almost all years except 2020.

### Hypertension (HTN) before pregnancy

**Fig 3:**
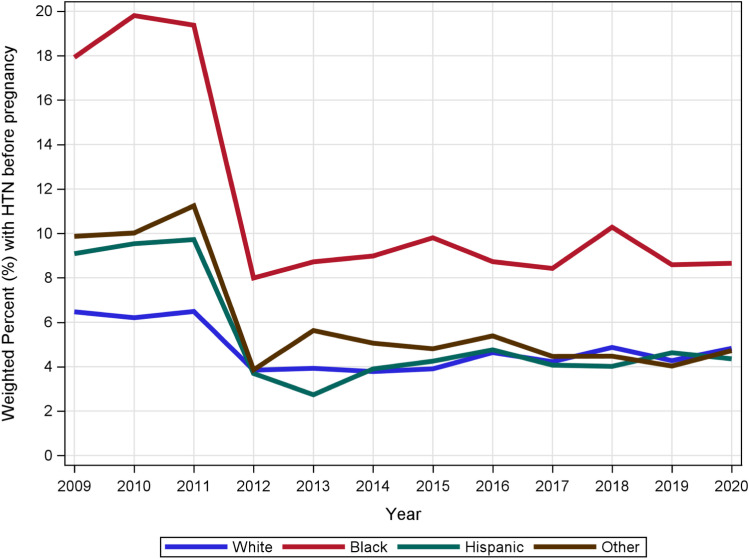
Race/ethnicity differences in hypertension before pregnancy over time.

Table 1 provides modeling for the impact of SLEs on HTN before pregnancy. Here, for every additional stressful life event, the risk of HTN before pregnancy increases by 8.2% (ARR = 1.082, 95% CI = [1.072, 1.093], *p* < 0.001). Effects of race/ethnicity depended upon year (*F* (33, 387,496) =3.42, *p* < 0.001), where Black women consistently had the greatest HTN before pregnancy prevalence (cf. Fig 3).

**Table 1:** Multivariable log-binomial modeling of hypertension before pregnancy.

Independent variable	ARR	95% CI for ARR	*P*-value
Stressful life events scale sum	1.082	1.072, 1.093	<.001
Race/Ethnicity			
Black	2.624	2.306, 2.987	<.001
Hispanic	1.397	1.154, 1.691	.001
Other	1.554	1.292, 1.869	<.001
White (RC)	–	–	–
Year × Race/Ethnicity			
2010 × Black	1.150	.955, 1.386	.141
2011 × Black	1.088	.903, 1.310	.377
2012 × Black	.768	.610,.967	.025
2013 × Black	.814	.649, 1.020	.074
2014 × Black	.849	.674, 1.069	.164
2015 × Black	.910	.742, 1.118	.370
2016 × Black	.702	.545,.904	.006
2017 × Black	.747	.601,.928	.008
2018 × Black	.830	.676, 1.018	.073
2019 × Black	.778	.627,.965	.022
2020 × Black	.650	.528,.801	<.001
2010 × Hispanic	1.083	.836, 1.402	.547
2011 × Hispanic	1.056	.829, 1.345	.659
2012 × Hispanic	.694	.502,.960	.027
2013 × Hispanic	.507	.371,.694	<.001
2014 × Hispanic	.740	.547, 1.001	.051
2015 × Hispanic	.779	.547, 1.110	.168
2016 × Hispanic	.751	.547, 1.031	.077
2017 × Hispanic	.723	.539,.970	.031
2018 × Hispanic	.580	.431,.781	<.001
2019 × Hispanic	.812	.593, 1.113	.195
2020 × Hispanic	.648	.468,.898	.009
2010 × Other	1.063	.819, 1.380	.644
2011 × Other	1.132	.888, 1.444	.316
2012 × Other	.662	.481,.911	.011
2013 × Other	.941	.703, 1.260	.682
2014 × Other	.868	.643, 1.173	.357
2015 × Other	.819	.602, 1.115	.205
2016 × Other	.791	.550, 1.138	.207
2017 × Other	.763	.558, 1.044	.091
2018 × Other	.617	.455,.836	.002
2019 × Other	.602	.432,.839	.003
2020 × Other	.712	.515,.985	.040

*Note. (ARR) adjusted relative risk ratio; Unweighted *n* = 350,948; Weighted *N* = 18,728,049. Additionally adjusting for year; year effects not shown (2009 = Reference category).

### Pregnancy-induced hypertension (PIH)

Due to limitations on collection the PIH data, the PIH analysis is limited to the seven states that have collected data all years. Subgroup analyses for the *seven states* collecting PIH from 2009–2020 revealed that SLE was related to PIH (ARR = 1.059, 95% CI= [1.043, 1.075], *p* < 0.001; Table 2), as well as race/ethnicity (*F* (3, 71,798) = 47.07, *p* < 0.001), and year (*F* (11, 71,790) = 6.37, *p* < 0.001). Here, after adjusting for year and SLE, Black women had 13.6% predicted prevalence of PIH (95% CI = [12.6%, 14.7%]) compared to White women (11.4%, 95% CI = [10.9%, 11.9%]), Hispanic women (7.6%, 95% CI = [6.9, 8.3%]), and other race/ethnicity (7.5%, 95% CI = [6.8%, 8.3%]).

**Table 2:** Multivariable log-binomial modeling of pregnancy-induced hypertension (Gestational Hypertension and Preeclampsia/Eclampsia).

Independent variable	ARR	95% CI for ARR	*P*-value
Stressful life events scale sum	1.059	1.043, 1.075	<.001
Race/Ethnicity			
Black	1.195	1.096, 1.303	<.001
Hispanic	.668	.604,.738	<.001
Other	.661	.591,.740	<.001
White (RC)	–	–	–

*Note. (ARR) adjusted relative risk ratio; (RC) reference category; Unweighted *n* = 65,774; Weighted *N* = 2,975,688. Additionally adjusting for year; year effects not shown.

### Gestational diabetes mellitus (GDM)

**Fig 4:**
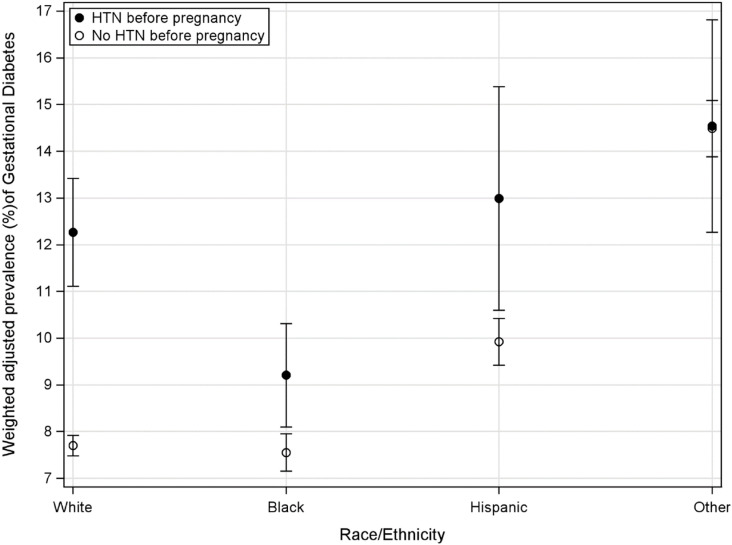
Model-based adjusted race/ethnicity differences in gestational diabetes. *Note HTN (Hypertension).

Finally, modeling results for GDM are presented in Table 3. SLE (ARR = 1.030, 95% CI=[1.020, 1.041], *p* < 0.001) as well as maternal age (ARR = 1.062, 95% CI=[1.059, 1.066], *p* < 0.001), education greater than 12 years versus 12 years or less (ARR = 0.883, 95% CI=[.843,.925], *p* < 0.001), overweight/obese BMI (ARR = 2.019, 95% CI=[1.938, 2.104], *p* < 0.001), private insurance vs. none (ARR = 0.867, 95% CI=[.822,.915], *p* < 0.001), any smoking during the 3^rd^ trimester (ARR = 1.090, 95% CI=[1.012, 1.173], *p* = 0.022), and interaction effects of race/ethnicity and HTN before pregnancy (*F*(3, 320,259) = 8.96, *p* < 0.001) were associated with GDM. To describe these interaction effects the adjusted predicted prevalence of GDM based on the multivariable modeling was plotted by race/ethnicity group and HTN before pregnancy status in Fig 4. The effect of having HTN before pregnancy on GDM substantially added risk for White mothers and, to a lesser degree, for Black and Hispanic mothers, but not for women of other races.

**Table 3:** Multivariable log-binomial modeling gestational diabetes.

Independent variable	ARR	95% CI for ARR	*P*-value
Maternal age (years)	1.062	1.059, 1.066	<.001
Education > 12 years	.883	.843,.925	<.001
Married vs. otherwise	1.023	.974, 1.075	.362
BMI overweight/obese vs. normal	2.019	1.938, 2.104	<.001
Below poverty income level	1.007	.950, 1.068	.808
Insurance			
Private	.867	.822,.915	<.001
Not Private	.977	.927, 1.029	.375
None (RC)	–	–	–
Used alcohol pre-pregnancy	.855	.822,.889	<.001
Any smoking during 3^rd^ trimester	1.090	1.012, 1.173	.022
Stressful life events scale sum	1.030	1.020, 1.041	<.001
Race/Ethnicity			
Black	.981	.922, 1.043	.537
Hispanic	1.289	1.213, 1.368	<.001
Other	1.881	1.790, 1.977	<.001
White (RC)	–	–	–
HTN before pregnancy	1.593	1.445, 1.756	<.001
Race/Ethnicity × HTN before pregnancy			
Black with HTN before pregnancy	.765	.651,.899	.001
Hispanic with HTN before pregnancy	.822	.665, 1.016	.070
Other with HTN before pregnancy	.630	.522,.760	<.001

*Note. (ARR) adjusted relative risk ratio; (HTN) hypertension; (RC) reference category; Unweighted *n* = 284,511; Weighted *N* = 15,481,949. *Not Private* insurance included Medicaid and state-specific SCHIP/CHIP/ and TRICARE. Additionally adjusting for year; year effects not shown.

## Discussion

We provided an overview of the trends of SLEs and their impacts on HTN before pregnancy, PIH, and GDM, by race/ethnicity using the national PRAMS data from 2009–2020. We found that the trends of SLEs from 2012 to 2015 were higher than those from 2016 to 2020. However, for Black women, SLE rates for almost all years were consistently high except in 2020. In addition, the risk of HTN before pregnancy increases by more than 8 percent for every additional SLE. SLEs were related to both HTN before pregnancy and PIH.

Previous studies from 1990 to 2011 on SLEs indicated a consistently high prevalence of SLE mothers [1,2,22]. In our study, we reported between 66% and 72% SLEs in women a year before a live birth from 2009 to 2020. These findings imply that the rates for SLEs have consistently been increasing for more than three decades. Even though the SLEs vary by year, the reductions in some years were not too different from those with higher values. Black women, however, have had consistently high SLEs except in 2020. While we cannot pinpoint the exact reasons for the reduction of SLEs among Black women in 2020, we can speculate that the events of 2020, which consisted of COVID-19 locked down, might have increased stress level for everyone or with social distance interventions, Black women were less likely to interact with SLEs triggers, thus lowering their stress level.

Our study SLEs was compared to HTN before pregnancy and PIH separately and GDM. We found that as the number of SLEs increases, the risk for HTN before pregnancy increases. SLEs were associated with PIH and GDM, which was further evident in Black women reporting the highest rate of HTN before pregnancy. In a previous study, Black women were more likely to report significant numbers of SLEs compared to other ethnicity/race [23]. In addition, SLEs during pregnancy were associated with hypertensive disorders of pregnancy and GDM [4,12,23].

### Implication of this study

The findings from this current study are based on many states, and the previous studies on one or a few states consistently found an increased trend of SLEs in women. With increasing adverse pregnancy outcomes, more attention on screening of SLEs in mothers may improve maternal outcomes. Future prospective studies need to investigate the relationship between SLEs and maternal complications to identify intervention target points to manage stress among mothers, especially before conception. In addition, more research is needed to understand the root cause of the consistent disparities in SLEs, potentially leading to understanding the disparities in adverse birth and maternal outcomes.

### Limitations and strengths

We analyzed data from states that met the required threshold and reported their data to the CDC. Additional data from the states below the threshold might differ, potentially leading to selection bias. However, the complex sampling design utilized in the Pregnancy Risk Assessment Monitoring System data ensures the generalizability of the results. However, the data analyzed is cross-sectional. For the analysis of PIH, we only included the seven states that reported on PIH consistently from 2019 to 2020. Additionally, the respondents self-reported HTN before pregnancy, PIH, and GDM. However, the self-report data were cross-checked with birth certificate records, a reliable source for prepregnancy and pregnancy maternal health information [24–26]. Finally, this study does not cover other factors that may contribute to adverse pregnancy outcomes, such as lack of prenatal care visits or lifestyle.

Despite the limitations, PRAMS is a national data with a large sample size that provides relevant updated literature on the trend of SLEs and their associations with HTN before pregnancy, PIH, and GDM from 2009 to 2020. Given that Black women continue to experience the highest SLEs and a higher risk of HTN before pregnancy and PIH, this report indicates there is an interplay among SLEs, HTN before pregnancy, PIH, and GDM, and race/ethnicity. Though the data are cross-sectional, they have been utilized to establish maternal behaviors and experiences before, during, and shortly after pregnancy. This study provided a literature update on the trend of SLEs and their associations with HTN before pregnancy, PIH, and GDM. This information is vital for public health efforts to reduce the disparities in adverse pregnancy outcomes.

## Conclusion

SLEs, as well as HTN before pregnancy, PIH, and GDM, are prevalent among mothers. These are also risk factors for cardiovascular disease. Additionally, Black women continue to experience the highest SLE and a higher risk of HTN before pregnancy and PIH, as well as poor maternal outcomes. Routinely screening and managing stress among women might mitigate the risk of pregnancy complications and their associated cardiovascular disease.

## References

[1] Burns ER, Farr SL, Howards PP, Centers for Disease Control and Prevention (CDC). Stressful life events experienced by women in the year before their infants’ births--United States, 2000-2010. MMWR Morb Mortal Wkly Rep. 2015;64(9):247–51. 25763877 PMC5779604

[2] Mukherjee S, Coxe S, Fennie K, Madhivanan P, Trepka MJ. Stressful life event experiences of pregnant women in the United States: a latent class analysis. Womens Health Issues. 2017;27(1):83–92. doi: 10.1016/j.whi.2016.09.007 27810166

[3] Whitehead NS, Brogan DJ, Blackmore-Prince C, Hill HA. Correlates of experiencing life events just before or during pregnancy. J Psychosom Obstet Gynaecol. 2003;24(2):77–86. doi: 10.3109/01674820309042805 12854392

[4] Chen L, Shi L, Chao MS, Tong X, Wang F. Stressful life events, hypertensive disorders, and high blood sugar during pregnancy. Stress Health. 2020;36(2):160–5. doi: 10.1002/smi.2911 31714017

[5] Venkatesh KK, Harrington K, Cameron NA, Petito LC, Powe CE, Landon MB, et al. Trends in gestational diabetes mellitus among nulliparous pregnant individuals with singleton live births in the United States between 2011 to 2019: an age-period-cohort analysis. Am J Obstet Gynecol MFM. 2023;5(1):100785. doi: 10.1016/j.ajogmf.2022.100785 36280146

[6] Liu SR, Glynn LM. The contribution of racism-related stress and adversity to disparities in birth outcomes: evidence and research recommendations. F S Rep. 2021;3(2 Suppl):5–13. doi: 10.1016/j.xfre.2021.10.003 35937456 PMC9349247

[7] Braveman P, Dominguez TP, Burke W, Dolan SM, Stevenson DK, Jackson FM, et al. Explaining the Black-White disparity in preterm birth: a consensus statement from a multi-disciplinary scientific work group convened by the March of Dimes. Frontiers in Reproductive Health. 2021:49. 10.3389/frph.2021.684207 PMC9580804 36303973

[8] Driscoll A, Gregory E. Prepregnancy body mass index and infant outcomes by race and Hispanic origin: United States, 2020. United States. 2021. 34982024

[9] Weber KA, Carmichael SL, Yang W, Tinker SC, Shaw GM, National Birth Defects Prevention Study. Periconceptional stressors and social support and risk for adverse birth outcomes. BMC Pregnancy Childbirth. 2020;20(1):487. doi: 10.1186/s12884-020-03182-6 32831042 PMC7446063

[10] Bryson CL, Ioannou GN, Rulyak SJ, Critchlow C. Association between gestational diabetes and pregnancy-induced hypertension. Am J Epidemiol. 2003;158(12):1148–53. doi: 10.1093/aje/kwg273 14652299

[11] Caplan M, Keenan-Devlin L, Freedman A, Grobman W, Wadhwa P, Buss C. Lifetime psychosocial stress exposure associated with hypertensive disorders of pregnancy. American journal of perinatology. 2021;38(13):1412–9. 32615616 10.1055/s-0040-1713368

[12] Morgan N, Christensen K, Skedros G, Kim S, Schliep K. Life stressors, hypertensive disorders of pregnancy, and preterm birth. J Psychosom Obstet Gynaecol. 2022;43(1):42–50. doi: 10.1080/0167482X.2020.1778666 32567962 PMC7865918

[13] Walker C, Kucharska-Newton A, Browning S, Christian W. County incidence and geospatial trends of early-onset hypertensive disorders of pregnancy in Kentucky, 2008-2017. BMC Pregnancy and Childbirth. 2023;23(1):1–9. 37337164 10.1186/s12884-023-05699-y PMC10278357

[14] Hackett RA, Steptoe A. Psychosocial factors in diabetes and cardiovascular risk. Curr Cardiol Rep. 2016;18(10):95. doi: 10.1007/s11886-016-0771-4 27566328 PMC5002050

[15] Dar T, Radfar A, Abohashem S, Pitman RK, Tawakol A, Osborne MT. Psychosocial stress and cardiovascular disease. Current treatment options in cardiovascular medicine. 2019;21:1–17. 31028483 10.1007/s11936-019-0724-5 PMC6568256

[16] Avorgbedor F, McCoy TP, Gondwe KW, Xu H, Spielfogel E, Cortés YI, et al. Cardiovascular disease-related emergency department visits and hospitalization among women with hypertensive disorders of pregnancy. Am J Prev Med. 2023;64(5):686–94. doi: 10.1016/j.amepre.2023.01.004 36863895 PMC11421440

[17] Chen L, Mayo R, Chatry A, Hu G. Gestational diabetes mellitus: its epidemiology and implication beyond pregnancy. Current Epidemiology Reports. 2016;3(1):1–11.

[18] Bellamy L, Casas J-P, Hingorani AD, Williams D. Type 2 diabetes mellitus after gestational diabetes: a systematic review and meta-analysis. Lancet. 2009;373(9677):1773–9. doi: 10.1016/S0140-6736(09)60731-5 19465232

[19] Shulman HB, D’Angelo DV, Harrison L, Smith RA, Warner L. The pregnancy risk assessment monitoring system (PRAMS): overview of design and methodology. Am J Public Health. 2018;108(10):1305–13. doi: 10.2105/AJPH.2018.304563 30138070 PMC6137777

[20] Williamson T, Eliasziw M, Fick GH. Log-binomial models: exploring failed convergence. Emerg Themes Epidemiol. 2013;10(1):14. doi: 10.1186/1742-7622-10-14 24330636 PMC3909339

[21] Graubard BI, Korn EL. Survey inference for subpopulations. American Journal of Epidemiology. 1996;144(1):102–6. 8659480 10.1093/oxfordjournals.aje.a008847

[22] Whitehead N, Hill HA, Brogan DJ, Blackmore-Prince C. Exploration of threshold analysis in the relation between stressful life events and preterm delivery. Am J Epidemiol. 2002;155(2):117–24. doi: 10.1093/aje/155.2.117 11790674

[23] Lawrence B, Kheyfets A, Carvalho K, Dhaurali S, Kiani M, Moky A, et al. The impact of psychosocial stress on maternal health outcomes: a multi-state PRAMS 8 (2016-2018) analysis. Journal of Health Disparities Research and Practice. 2022;15(2):7–7.

[24] Dietz P, Bombard J, Mulready-Ward C, Gauthier J, Sackoff J, Brozicevic P, et al. Validation of self-reported maternal and infant health indicators in the pregnancy risk assessment monitoring system. Matern Child Health J. 2014;18(10):2489–98. doi: 10.1007/s10995-014-1487-y 24770954 PMC4560102

[25] Farley KE, Huber LRB, Warren-Findlow J, Ersek JL. The association between contraceptive use at the time of conception and hypertensive disorders during pregnancy: a retrospective cohort study of prams participants. Matern Child Health J. 2014;18(8):1779–85. doi: 10.1007/s10995-014-1447-6 24535145

[26] Vinikoor LC, Messer LC, Laraia BA, Kaufman JS. Reliability of variables on the North Carolina birth certificate: a comparison with directly queried values from a cohort study. Paediatr Perinat Epidemiol. 2010;24(1):102–12. doi: 10.1111/j.1365-3016.2009.01087.x 20078836 PMC3437766

